# The effects of hydrolysis condition on antioxidant activity of protein hydrolyzate from *quinoa*

**DOI:** 10.1002/fsn3.871

**Published:** 2019-02-10

**Authors:** Mina Mahdavi‐Yekta, Leila Nouri, Mohammad Hossein Azizi

**Affiliations:** ^1^ Department of Food Science and Technology Damghan Branch Islamic Azad University Damghan Iran; ^2^ Department of Food Science and Technology School of Agriculture Tarbiat Modares University Tehran Iran

**Keywords:** DPPH radical scavenging, enzymatic hydrolysis, protein hydrolyzate, quinoa

## Abstract

In the present study, *quinoa* protein hydrolyzate was prepared using alcalase and pepsin enzymes. Then, the effect of different temperatures (40, 45, 50, and 55°C), periods of time (60, 120, 150, 180, and 210 min), and the ratio of enzyme to substrate (30, 60, and 90 Anson unit/kg protein) on degree of hydrolysis were examined. Also, the antioxidant activity was assessed using DPPH radical scavenging test, and investigated using a completely randomized design. According to results, the optimum condition to produce hydrolyzates with the highest degree of hydrolysis (24.65%) was 55°C, 210 min, with ratio of enzyme to substrate of 60 Anson unit/kg protein, The highest antioxidant activity (35.44) of protein hydrolyzed was achieved at 150 min, 50°C, and the ratio of enzyme to substrate 60 (Anson unit/kg protein). Moreover, there was no significant (*p *> 0.05) between the level of hydrolysis and the antioxidant activity was among different time and temperatures. In conclusion, the peptide derived from *quinoa* protein showed a sufficient antioxidant activity to be incorporated in food products.

## 1 INTRODUCTION

Lipid oxidation and auto‐oxidation as two major issues could induce deteriorative changes regarding the quality and safety of food products (Hashemi et al., 2016, 2017a,2017b; Morrissey, Sheehy, Galvin, Kerry, & Buckley, 1998). Synthetic antioxidants such as butylated hydroxytoluene, butylated hydroxyanisole, and tertiary butylhydroquinone have been used to control the lipid oxidation process (Maqsood, Benjakul, Abushelaibi, & Alam, 2014). However, due to potential toxicity and adverse effects on human health, their incorporation in the formulation of food raised notable concerns (Pateiro et al., 2018). In this regard, different sources including animal species and plant sources were investigated to discover new natural preservatives.

Quinoa as a flowering plant with high nutritious value belongs to the amaranth family (Nongonierma, Le Maux, Dubrulle, Barre, & FitzGerald, 2015). It has been traditionally cultivated in the Andean region of northwestern South America since thousands of years ago (Vilcacundo, Martínez‐Villaluenga, & Hernández‐Ledesma, 2017). Quinoa and its derived products contain both macronutrients and micronutrients, for example, protein, polysaccharides, fatty acids, fiber, polyphenols, vitamins, and minerals (Park, Lee, Kim, & Yoon, 2017). The higher protein concentrations of quinoa as a gluten‐free grain while compared with other dietary grains such as wheat, rice, maize, oat, and barley are well documented (Calderelli, Benassi, Visentainer, & Matioli, 2010; Comai et al., 2007; Nongonierma et al., 2015). Several investigations were carried out to evaluate the functional properties of quinoa, including treatment of hypertension, diabetes, hypercholesterolemia, and celiac disease (CD) (Asao & Watanabe, 2010; Vilcacundo et al., 2017; Zevallosm, Herencia, & Ciclitira, 2013). In this regard, according to Food and Agriculture Organization (FAO), quinoa is an ancient crop with notable contribution in world food security (Rizzello et al., 2017).

Over the last few years, the use of natural antioxidants has attracted particular attention, and in this regard, the application of peptides derived from hydrolyzed proteins obtained from soy, wheat, milk casein, and fish due to their antioxidant activities attracted notable attention (Calderelli et al., 2010; Rizzello et al., 2017). The antioxidants could be obtained via a wide spectrum of biological techniques such as protein hydrolysis, as a highly qualified technology used to produce high‐quality products (Samaranayaka & Li‐Chan, 2011; Sarmadi & Ismail, 2010). According to the literature, the biologically active peptides with potential antioxidant activity can be derived from a variety of animal‐ or plant‐derived protein sources, including rice bran, sunflower protein, corn gluten meal, egg‐yolk protein, mushroom, peanut kernels, buckwheat protein, milk kefir, and soy milk kefir (Rizzello et al., 2017; Sarmadi & Ismail, 2010). Among the produced antioxidant agents, the antioxidant and antimicrobial activities of Quinoa compounds have been investigated in some studies (Gorinstein et al., 2008; Miranda et al., 2014; Park et al., 2017; Tang et al., 2015). However, quinoa proteins as a source of bioactive peptides were mentioned in few of them. In this context, the bioactive peptides are defined as specific amino acid sequences which enhance some useful biological activities (de Castro & Sato, 2014). Bioactive peptides can be defined as specific protein fragments with potential biological activities. Based on their structure, composition, and sequence, they may demonstrate antioxidative, antihypertensive, and antibacterial bioactivities and even reduce the cholesterol levels (Harnedy & FitzGerald, 2012; Nasri et al., 2014).

Trypsin, chymotrypsin, pepsin, and alcalase as proteolytic plant/microbe‐derived enzymes are used in the food industry for proteins hydroxylation (Sumantha, Larroche, & Pandey, 2006; Tavano, 2013). Protein hydrolysis with the aid of these enzymes is the most broadly used method in the production of biologically active peptides. Based on our knowledge, no investigation was conducted regarding the application of proteolytic enzymes in the production of protein hydrolyzates from quinoa. Therefore, the current study was undertaken to produce protein hydrolyzates from the Quinoa by using pepsin and alcalase and also the antioxidant activity of protein produced of hydrolyzates from quinoa was evaluated.

## 2 MATERIALS AND METHODS

### 2.1 Sample preparation

The quinoa seeds (Santa Maria variety) were obtained from The Karaj Seed and Plant Improvement Institute, Karaj, Iran, in autumn of 2017. Grain mill was produced out at the Karaj seed and Plant Improvement Institute using a hammer mill (screen sizes 3.18 mm, GIDC, Ahmedabad, Gujarat, India), and a Whole‐quinoa flour with a degree of extraction of 96% was obtained. In order to extract of quinoa fat, the produced flour was mixed with hexane as solvent in a ratio of 1:5 among 24 hr with the aid of a fattened shaker (Fisher Scientific Ltd, cat. no.14‐285‐729). Afterward, the fat‐free flour was placed for 24 hr in an oven at 40°C, to isolate the residues of solvent, and to obtain a good powdered flour. The resulting flour was sieved using 0.25‐mm mesh and then was kept in polyethylene bags in a freezer at −18°C until the time of the experiment (Nongonierma et al., 2015).

### 2.2 Extraction of Quinoa protein

The recommended method by Chauhan, Cui, and Eskin (1999) with some modifications was used to extract the protein concentrate from the quinoa seed flour. Accordingly, the flour of quinoa was dispersed in a solution of sodium hydroxide (0.015 M). The resulting slurry was kept for 24 hr at 4°C to improve the clarity of supernatant and then was centrifuged (Sigma, 6k15, Germany) at 10,000 *g*, 10°C for 30 min. After that, the supernatant was filtered using a Whatman paper (Whatman No. 1), and the pH value of the filtrate was adjusted to 4.5 by addition of 0.1 N HCl for precipitating the proteins. The precipitated part of the proteins was isolated through the 30‐min centrifugation (Sigma, 6k15) at 10,000 *g*, 10°C. After that, it was washed with distilled water and then was lyophilized (freeze dryer, alpha 2, Christ‐Germany) to produce quinoa protein concentrate. The analysis of quinoa protein concentrate was carried out according to American Association of Cereal Chemists (Method 46‐19, AACC 1983). The nitrogen amount was converted to protein content by a factor of 5.7 according to the previous studies (Fujihara, Kasuga, & Aoyagi, 2001; Mariotti, Tomé, & Mirand, 2008).

### 2.3 Protein hydrolysis

The proteins extracted from quinoa were digested using pepsin and alcalase enzymes in a glass container with the aid of a magnetic agitator. The slurry of protein concentrates with the concentration of 5% in distilled water w/v (based on the protein content) was prepared, and the pH of solution was set as 8.0 using NaOH 2 M (The appropriate temperature for alcalase enzyme activity). Subsequently, the protein slurry was heated to 50°C and then was charged with enzyme alcalase at a concentration of 4% (w/w, quinoa protein basis) under a mild condition of stirring. The samples were hydrolyzed at 50°C for 4 hr while the pH value was kept constant by adding the sodium hydroxide solution (2 M). Then, pH of the mixture was reached to 2.5 and the solution was uploaded to be digested with pepsin enzyme. After digestion, they were boiled in water for 15 min to discover the optimized hydrolysis optimization condition. Afterward, the samples were cooled to the room temperature using cold water and centrifuged (Sigma, 6k15) at 10,000 *g*, 10°C for 15 min, and a portion of the supernatant (protein hydrolyzates) was lyophilized by freeze dryer (freeze dryer, alpha 2, Christ‐Germany) (Toapanta, Carpio, Vilcacundo, & Wilman, 2016).

The method which was described by Nongonierma et al. (2015) was utilized to determine the degree of hydrolysis (DH %). The residual portion of the protein hydrolyzates was passed over Amicon stirred cell ultrafiltration setup (Fisher Scientific, ON, Canada) with the aid of a membrane (molecular weight cutoff of 10,000 or 5000 Da). In each step, a semiquantitative separation column C‐18 was used to perform the chromatogram analysis on the hydrolyzate. The permeate obtained from each membrane was lyophilized and kept at −20°C until day of the experiment.

The method of Le Maux, Nongonierma, Murray, Kelly, and FitzGerald (2015) was employed to measure the protein content of permeates and hydrolyzates. To find the appropriate range of hydrolysis optimization conditions, the pretreatments were performed at 40, 45, 50, 55°C, 60, 90, 150, 180, and 210 min and 30, 60, and 90 Anson unit/kg protein.

### 2.4 DPPH radical scavenging test

The capacity of the samples to scavenge the DPPH‐free radicals was measured using a previously ascribed technique (Bey, Louaileche, & Zemouri, 2013). In this context, 500 μl of each sample was charged with 500 μl of ethanol (99.5%) and 25 μl of DPPH (0.22%) in ethanol (99.5%), then vigorously mixed, and then was stored for 30 min at a dark place. The sample‐free reagent was also used as the control. The absorbance was determined spectrophotometrically at 517 nm with a UV–vis spectrophotometer (DR 5 000^™^ UV–Vis Spectrophotometer). Finally, the DPPH radical scavenging capacity of the specimens was calculated with the help of the following equation.

(1) $$\;\text{DPPH radical scavenging activity}\;\left(\%\right)=\frac{\text{Absorb blank sample}-\text{Absorb control}}{\text{Absorb control}}\times 100$$

### 2.5 The degree of hydrolysis (DH) measurement

The DH was measured based on the previously conducted method (Lambers et al., 2015), to measure the percentages of soluble proteins in 10% trichloroacetic acid to the total proteins in the sample. In this regard, 5 ml of the sample was mixed with 5 ml of trichloroacetic acid (10%) followed byentrifuging at 10,000 *g*, 10°C for 20 min. Then, the concentration of protein was measured by Kjeldahl method, and the degree of hydrolysis was determined according to the following equation (Lambers et al., 2015).

(2) $$\text{The}\;\text{degree}\;\text{of}\;\text{hydrolysis}\;\left(\text{DH}\%\right)=\frac{\text{Nitrogen}\;\text{soluble}\;\text{in}\;\text{trichloroacetic}\;\text{acid}\;10\%}{\text{Total}\;\text{nitrogen}\;\text{of}\;\text{sample}}\times 100$$

### 2.6 Statistical analysis

SPSS software (ver. 19.0) was employed in this study. All the experiments were repeated in triplicate. Data of study were analyzed using two‐way analysis of variance (ANOVA), and results were expressed as mean ± standard deviation. Differences were considered significant at *p* < 0.05.

## 3 RESULTS AND DISCUSSION

### 3.1 Evaluate progress of hydrolysis

**Figure 1:**
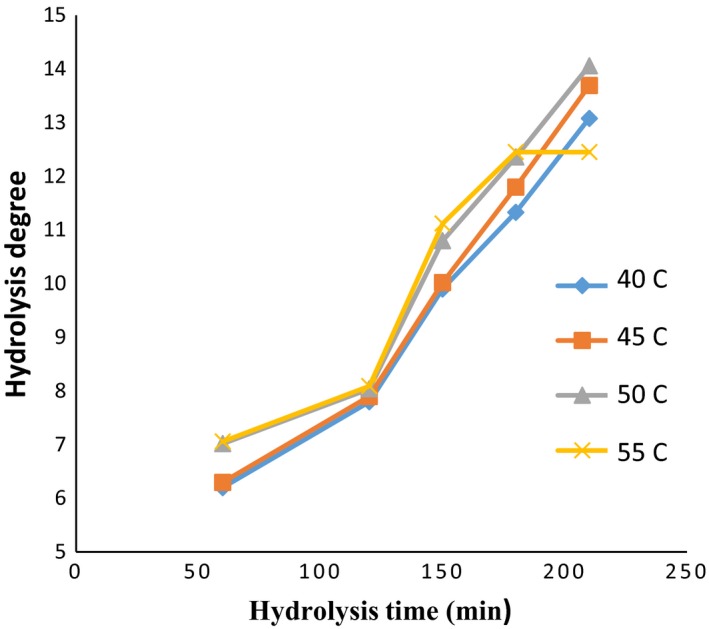
DE values: in the ratio of enzyme to substrate (30 Anson unit/kg protein) (alcalase–pepsin) at different times and temperatures

**Figure 2:**
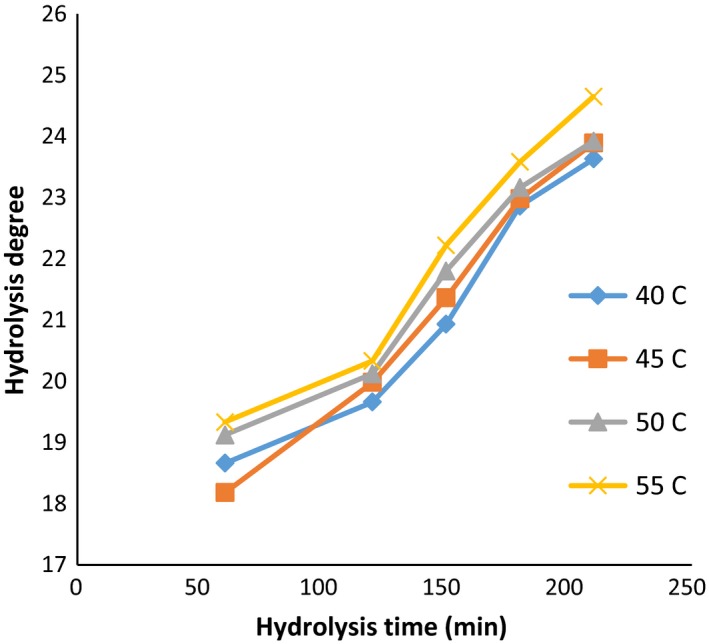
DE values: in the ratio of enzyme to substrate (60 Anson unit/kg protein) (alcalase–pepsin) at different times and temperatures

**Figure 3:**
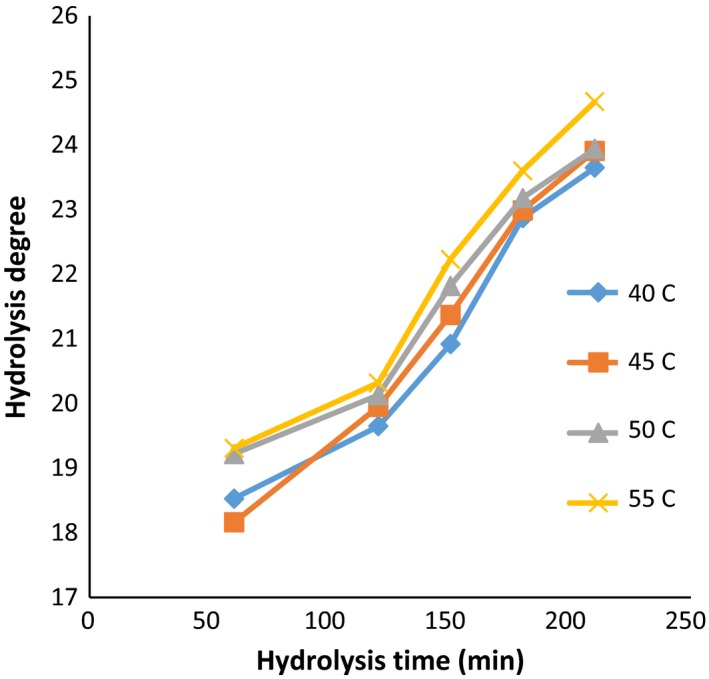
DE values: in the ratio of enzyme to substrate (90 Anson unit/kg protein) (alcalase–pepsin) at different times and temperatures

Further control in the rate of hydrolysis during the hydrolysis process is crucial due to its effects on the properties of the hydrolyzed protein, including free amino acids, the solubility, the molecular weight of the resulting peptides, and even the oxidative properties of the protein produced (Šližytė, Daukšas, Falch, Storrø, & Rustad, 2005). The influence of the enzyme activity and hydrolysis temperature on the degree of hydrolysis is documented through Figures 1, 2, 3.

Moreover, the factor of time has a significant effect on the DH (*p *< 0.05). In general, with increasing in hydrolysis time, the DH was increased. The highest value of DH among all temperatures and enzyme activity was achieved at 210 min. However, it was noted that hydrolysis degree obtained in treatments of 180 and 210 min was not significantly different. The lowest degree of hydrolysis was nominated to time of 60 min.

The highest DH (24.65%) was obtained at the temperature of 55°C, with hydrolysis time of 210 min, while the enzyme ratio was adjusted as 60 (Anson unit/kg protein). In this context, the temperature factor also showed a significant effect on the DH (*p* < 0.05). However, no significant difference was observed between 45 and 50°C due to higher mean hydrolysis rates at 55°C, and the temperature of 55°C was determined as optimized temperature. According to Ovissipour, Taghiof, Motamedzadegan, Rasco, and Molla (2009) with increasing in the time and the concentration of enzyme, the DH value was increased.

Enzyme activity also showed a significant effect on the degree of hydrolysis (*p *< 0.05). Considering to findings, a notable increase in the degree of hydrolysis was achieved by increasing in the enzyme activity from 30 to 90 (Anson unit/kg protein). The difference in degrees of hydrolysis between enzyme activities 60 and 90 (Anson unit/kg protein) was less than the difference observed among increasing in 30–60. However, no significant difference between enzyme activity of 60 and 90 (Anson unit/kg protein) at 50°C was noted which might be correlated with the saturation between enzyme concentration and substrate. Probably the higher degree of hydrolysis by increasing in the enzyme concentration can be attributed to higher activity of enzyme and consequently the breakdown of more peptide bands (Ambigaipalan, Al‐Khalifa, & Shahidi, 2015; Bougatef et al., 2010).

Generally, the denaturation of proteins can be used as an efficient pretreatment to enhance the performance of enzymatic cleavage. Nevertheless, the breaking of more peptide bonds results in the destabilization of the protein molecule, and as a consequence, smaller peptide units are produced. It was well documented that the difference in the rate of the peptide cleavage was associated with hydrolysis factors such as temperature, time, pH value, and the concentration of enzyme (Kristinsson & Rasco, 2000; Saidi, Belleville, Deratani, & Amar, 2013; See, Hoo, & Babji, 2011). Accordingly, variety of optimum hydrolysis conditions can be proposed for various substrates based on the type of the substrate, particularly the amount and reactivity of any endogenous proteases. In this regard, according to Bhaskar and Mahendrakar (2008), the optimum conditions for producing hydrolyzates with a high degree of hydrolysis (about 50%) with the action of alcalase were ascribed as following: the enzyme‐to‐substrate ratio of 1.5% (v/w), the temperature of 50°C, and the hydrolysis time of 135 min. However, Guerard, Guimas, and Binet (2002) suggested that the decrease in the hydrolysis degree by increasing the time of hydrolysis can be correlated with further limitations in enzyme activity through the generation of reaction products at high degree of hydrolysis, reduction in peptide bond concentration available for the hydrolysis, enzyme inhabitation, and deactivation of the enzyme. Therefore, it can be concluded that the polypeptide chains of *quinoa* protein were highly available for enzymatic cleaving at the higher hydrolysis time (210 min) and temperature (55°C). Concerning enzyme concentration, the DH value was increased by increasing the concentrations (60 and 90 Anson unit) due to the availability of higher enzyme molecules.

### 3.2 Inhibition of DPPH‐free radicals

**Figure 4:**
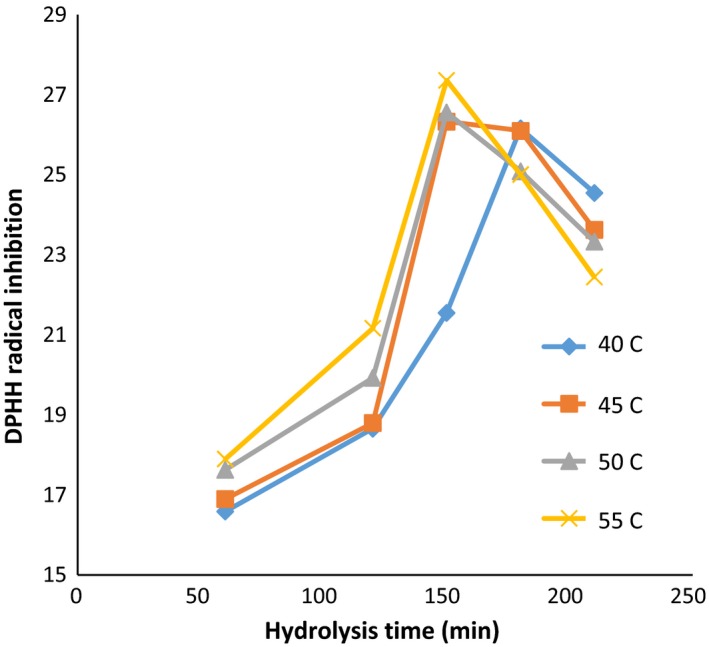
DPPH values: in the ratio of enzyme to substrate (30 Anson unit/kg protein) (alcalase–pepsin) at different times and temperatures

**Figure 5:**
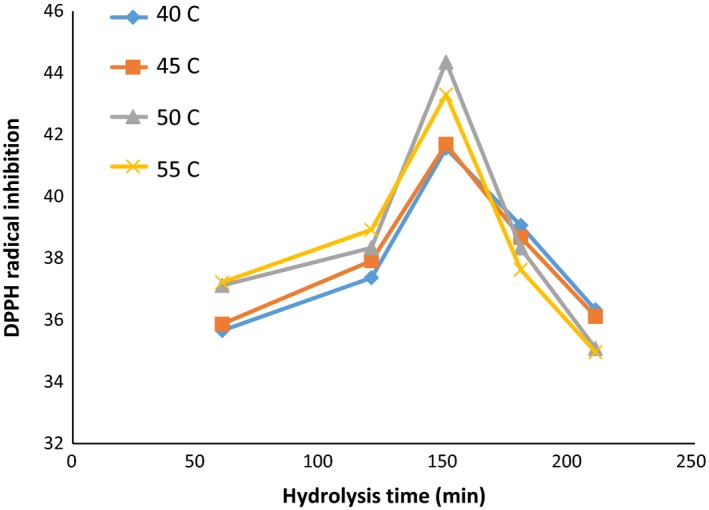
DPPH values: in the ratio of enzyme to substrate (60 Anson unit/kg protein) (alcalase–pepsin) at different times and temperatures

**Figure 6:**
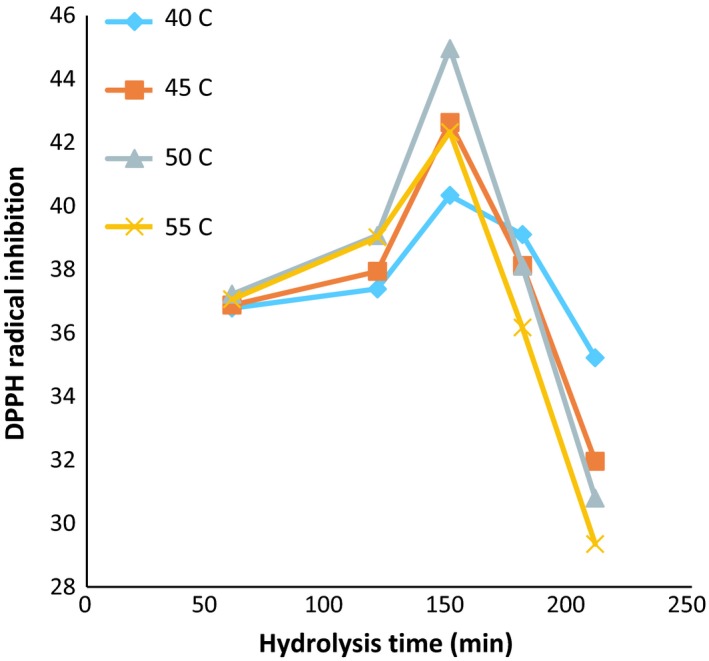
DPPH values: in the ratio of enzyme to substrate (90 Anson unit/kg protein) (alcalase–pepsin) at different times and temperatures

The variation in inhibitory characteristics of DPPH free radicals in different times, temperatures, and enzymes concentration is shown in Figures 4, 5, 6. Table 1 is also presented for comparison between study groups (DPPH‐DH). The highest activity of inhibiting DPPH radicals (35.44) was achieved after 150 min, at 50°C, and the ratio of enzyme to the substrate of 60 (Anson unit/kg protein). However, there was no significant difference in the DH between different time, temperature, and ratios of enzyme; 60 and 90 (Anson unit/kg protein); and the activity of inhibition of DPPH‐free radicals was observed (*p *> 0.05).

**Table 1:** Comparing the study groups in multiple aspects^a^

	Differences	Low limit	High limit	*p*‐Value^b^
DPPH‐DH (ratio of alcalase enzyme: 30 Anson unit/kg protein)	12.3	2.46	24.02	<0.05
DPPH‐DH (ratio of alcalase enzyme: 60 Anson unit/kg protein)	10.4	0.98	23.14	<0.05
DPPH‐DH (ratio of alcalase enzyme: 90 Anson unit/kg protein)	8.7	0.46	14.76	<0.05

a Multiple Tokyo HSD comparison test.

b Significant difference at 5% error level.

As shown in Figure 5, with increasing in hydrolysis time in all activities of the enzyme 30, 60, and 90 (Anson unit/kg protein), the antioxidant activity increased at 120 and 150 min and then followed a downward trend. Reducing the amount of inhibitory activity of hydrolyzed protein by increasing in the hydrolysis time can be correlated with the progression of the amount of hydrolysis and the higher effects of the enzyme on the protein substance, and also breaking in some of the antioxidant peptides formed in the early stages of hydrolysis.

The differences in the size and amount as well as the structure of peptides and amino acids during hydrolysis could influence the antioxidant activities (Wu, Chen, & Shiau, 2003), while the quenching of the free radicals was suggested as the main mechanism for this property (Peng, Xiong, & Kong, 2009). Zhidong et al. (2013) stated that by increasing in the hydrolysis time and enzyme ratio, the antioxidant capacity showed an increasing trend following with a decline. According to their findings, the optimized condition of hydrolysis for whey protein isolate was summarized as enzyme‐to‐substrate ratio of 2.22% (W/W), hydrolysis time of 3.60 hr, and the temperature of 45.70°C. Also, increasing in the time of hydrolysis caused the gradual improvement in DPPH‐free radical scavenging ability (Wu et al., 2003). Moreover, the antioxidant capacity of the hydrolyzates prepared from camel milk caseins significantly improved by increasing in the hydrolysis time and also the DH value (Kumar, Chatli, Singh, Mehta, & Kumar, 2016). Likewise, the activity to scavenge the DPPH radicals of yak milk protein hydrolyzates (produced by Alcalase) was enhanced with the progressive increase in the hydrolysis process up to 7 hr (Mao, Cheng, Wang, & Wu, 2011). Also, the antioxidant activity of the enzymatic hydrolyzates produced from round scad muscle protein (by alcalase) and brown stripe red snapper muscle (by the action of flavourzyme) was improved by increasing in the DH value (Khantaphant, Benjakul, & Kishimura, 2011; Thiansilakul, Benjakul, & Shahidi, 2007). In the contrast, it was reported that the DPPH radical scavenging activity of the bovine casein hydrolyzates (produced with different proteolytic enzymes) was lower compared to the native parental proteins (Rival, Boeriu, & Wichers, 2001).

## 4 CONCLUSION

The results of an investigation of the antioxidant properties of hydrogenated proteins from quinoa showed the production of this product is effectively influenced by the reaction conditions, namely, temperature, hydrolysis time, and enzyme activity. In fact, each of the factors had an impact on the antioxidant features of the peptides. Finally, it can be said that quinoa can be used as a suitable source for the production of active antioxidant peptides as a natural preservative in food formulations.

## CONFLICT OF INTEREST

The authors declare that they have no conflict of interest.
